# Relationship between cortisol and diabetic microvascular complications: a retrospective study

**DOI:** 10.1186/s40001-023-01325-x

**Published:** 2023-09-29

**Authors:** Shengnan Sun, Yangang Wang

**Affiliations:** 1 The Affiliated Hospital of Qingdao University, Qingdao, China; 2 Tianjin Medical University, Tianjin, 300134 China

**Keywords:** Type 2 diabetes, Diabetic microvascular complication, Cortisol, Logistic regression analysis

## Abstract

**Objective:**

We aimed to investigate whether serum cortisol associate with diabetic microvascular compliments in patients with type 2 diabetes mellitus (T2DM).

**Materials and methods:**

The subjects were recruited from hospitalized patients with T2DM from 2019 to 2021. The odds ratios (OR) and corresponding 95% confidence intervals (CI) in relation to cortisol quartiles were obtained by multiple logistic regression analysis.

**Results:**

(1) Cortisol level was positively correlated with the severity of microalbuminuria. The OR (95% CI) of microalbuminuria and macroalbuminuria in the last quartile were 3.396 (2.030, 5.682) and 8.407 (3.726, 18.971) compared with the first quartile (*p* < 0.001). (2) Cortisol level was positively correlated with the severity of diabetic retinopathy (DR). The OR (95% CI) of non-proliferative diabetic retinopathy group (NPDR) and proliferative diabetic retinopathy group (PDR) in the last quartile were 2.007 (1.401, 2.875) and 7.122 (2.525, 20.090) compared with the first quartile. (3) Elevated cortisol level was associated with diabetic peripheral neuropathy. The OR (95% CI) of diabetic peripheral neuropathy (DPN) in the last quartile was 1.956 (1.371, 2.792) and that in the third quartile was 1.854 (1.319, 2.608).

**Conclusions:**

High serum cortisol levels were significantly associated with diabetic microvascular compliments in inpatients. Its causality remains to be further studied.

*Clinical trial registration number*: ChiCTR2100051749.

## Introduction

Microvascular complications of T2DM include diabetic kidney disease (DKD), DR and DPN. DKD is the major cause of renal failure, and microalbuminuria excretion increased in patients with T2DM from 30 to 50% [1, 2]. As the worldwide prevalence of diabetes mellitus continues to increase, DR remains as leading cause of vision loss and preventable blindness in adults [3, 4]. DPN is the most common chronic complication of type 1 diabetes mellitus (T1DM) and T2DM. It will increase the risk of infection, foot ulcers and amputation, and about 50% of diabetic patients will eventually develop DPN [5]. Diabetic microvascular complications are particularly serious manifestation in the progression of diabetes. Consequently, early identification of diabetic microvascular complications might contribute to prevent the progression of T2DM.

Cortisol is a glucocorticoid secreted by adrenal cortex, which plays an important role in glucose, fat and protein metabolism. Hypercortisolism is associated with a variety of diseases, including diabetes, obesity, hypertension, osteoporosis and cardiovascular disease [6]. The prevalence of hypercortisolism in diabetic patients was higher than that in the general population [7, 8]. Study indicated that cortisol was positively correlated with microalbuminuria in T2DM [9]. However, it only emphasized patients with microalbuminuria and the sample size was small. A small cross-sectional study suggested that cortisol might be a molecular marker for the severity of DR [10]. The study also had a small sample size. Hence, this study deemed it necessary to explore the relationship between cortisol and diabetic microvascular complications in T2DM. For this reason, we retrospectively investigated the potential association between serum cortisol levels and diabetic microvascular complications, especially DKD, DR and DPN. Compared to previous literature, our study had an adequate sample size and focused on both patients with microalbuminuria and macroalbuminuria. This study aimed to emphasize the role of cortisol in the progression of diabetic microvascular complications, and to evaluate cortisol as a clinical marker for identifying and staging diabetic microvascular complications.

## Materials and methods

### Study population

The subjects were recruited from hospitalized patients with T2DM from 2019 to 2021. Analysis data is collected from the clinical data platform of the Affiliated Hospital of Qingdao University, which is distributed in four regions in Qingdao, Shandong. The inclusion criteria accorded with the American Diabetes Association (ADA) in 2014 [11]: HbA_1_c ≥ 6.5%, or fasting plasma glucose (FPG) ≥ 126 mg/dL (7.0 mmol/L),or Two-hour plasma glucose ≥ 200 mg/dL (11.1 mmol/L) during an OGTT, or a random plasma glucose ≥ 200 mg/dL (11.1 mmol/L) with classic symptoms of hyperglycemia or hyperglycemic crisis. Participants aged < 18 years or > 80 years, with acute complications of T2DM, acute infection, history of hormone use, hypercortisolism, history of drugs affecting the HPA axis, autoimmune disease, severe heart failure, severe liver disease, renal insufficiency requiring dialysis, and malignant tumors were excluded. Pregnant or lactating women were also excluded. All patients received diabetic dietary instructions while in hospital, which ensured that all patients had a similar dietary composition. Patients with poor compliance in this study will be excluded. The program was designed according to the declaration of Helsinki and approved by the ethics committee of the Affiliated Hospital of Qingdao University. All participants provided written informed consent. The study is registered on http://www.chictr.org.cn/ under the registration number ChiCTR2100051749.

### Anthropometric and laboratory data

Data collection was carried out by professional staff according to standard guidelines. The anthropometric parameters of the patient included age, height, weight, waist circumference and hip circumference, duration of diabetes, drinking and smoking, and blood pressure. Through laboratory examination, we measured the following indexes: fasting blood glucose, glycosylated hemoglobin, cortisol, low density lipoprotein cholesterol (LDL-C), high density lipoprotein cholesterol (HDL-C), free fatty acid (FFA), triglyceride (TG), total cholesterol (TC), serum creatinine (CR) and serum uric acid (UA). Blood pressure (BP) was measured after five minutes of rest and averaged for two consecutive days or more. Body mass index (BMI) was calculated as weight divided by height squared (kg/m^2^). Blood samples were taken at 8 a.m. after fasting for at least 8 h. Blood samples were obtained by puncturing the median elbow vein, stored at low temperature and centrifuged within 1 h, and transported to the central laboratory for testing as soon as possible. Plasma cortisol was determined by electrochemiluminescence immunoassay. HbA1c was determined by high performance liquid chromatography (MQ-2000PT, China). Blood glucose and lipids were measured by Beckman Coulter Au 680 (Germany). UA was measured by DIMENSION LXR automatic analyzer. Serum creatinine was measured by picric acid method (Coulter Au 680). Urinary creatinine was measured by enzyme method, and urinary microalbumin (AU 680) was measured by immunoturbidimetry. eGFR was calculated according to the collaborative epidemiology of chronic kidney disease (CKD-EPI) formula.

On the grounds of the classification suggested in the kidney disease: improving global outcomes guidelines (KDIGO), the categories of proteinuria were defined: normal albuminuria (UACR < 30 mg/g), microalbuminuria (UACR 30–299 mg/g) and macroalbuminuria (UACR ≥ 300 mg/g) [12, 13]. After blood samples were collected, retinopathy was evaluated by funds camera (AFC-330, NIDEX, Japan), slit lamp microscope (3020H, Keeler Ltd, United Kingdom) and noninvasive optical coherence tomography (5000, Carl Zeiss, United States of America). According to the definition of Wilkinson et al. [11], the patients were divided into three groups: non diabetic retinopathy group (NDR), NPDR and PDR. DPN is defined as clinical and electrophysiological evidence of the definite presence of peripheral neuropathy in patients with diabetes. Well trained staff conducted standard physical examination for patients, including ankle reflex, vibration sense, pressure sense, acupuncture pain sense and temperature sense. Clinical diagnostic criteria for DPN: (1) neuropathy at the time of or after the diagnosis of diabetes. (2) Clinical symptoms of neuropathy, such as pain, numbness, abnormal sensation, etc., and abnormalities in any 1 of the 5 tests (ankle reflex, vibration sensation, pressure sensation, temperature sensation, pinprick pain sensation); if there are no clinical symptoms, abnormalities in any 2 of the 5 tests may also be diagnosed. (3) Neuropathy due to other causes. Apart from that, all participants were evaluated by electromyography to assess motor nerve conduction velocity, sensory nerve conduction velocity and sympathetic skin response [14, 15]. It is common for patients with DPN to present with abnormal nerve conduction velocity.

### Statistical analysis

The PASS version 15.0 software (NCSS, Kaysville, Utah, USA) was used to calculate the sample size. When the expected mean (SD) of cortisol in the normoalbuminuria, microalbuminuria and macroalbuminuria group was defined as 260(80), 280(100) and 300(120) respectively, a sufficient power over 90% need at least 855 individuals in DKD group. Similarly, we calculated that at least 799 and 736 subjects were needed for the DR and DPN group. SPSS version 24.0 software (SPSS IBM, Armonk, New York, USA) was used for statistical analysis. Continuous variables of normal distribution were expressed as mean ± standard deviation (SD), while continuous variables of non-normal distribution were expressed as interquartile range (IQR), and classified variables are expressed as frequency. Chi square test or Kruskal Wallis test were used to compare categorical variables and continuous variables. The mean score of plasma cortisol level was divided into quartiles. The first quartile (Q1) represented the lowest quartile and the fourth quartile (Q4) represented the highest quartile. Multiple logistic regression analysis was performed to obtain odds ratio (OR) and corresponding 95% confidence interval (CI). Receiver operating characteristic (ROC) analyses were performed to detect the optimum cutoff value, which was calculated by the sum of sensitivity and specificity in the ROC curve. The value of *p* < 0.05 was considered statistically significant (two sided).

## Results

### Association of cortisol level with microalbuminuria and macroalbuminuria

**Fig. 1:**
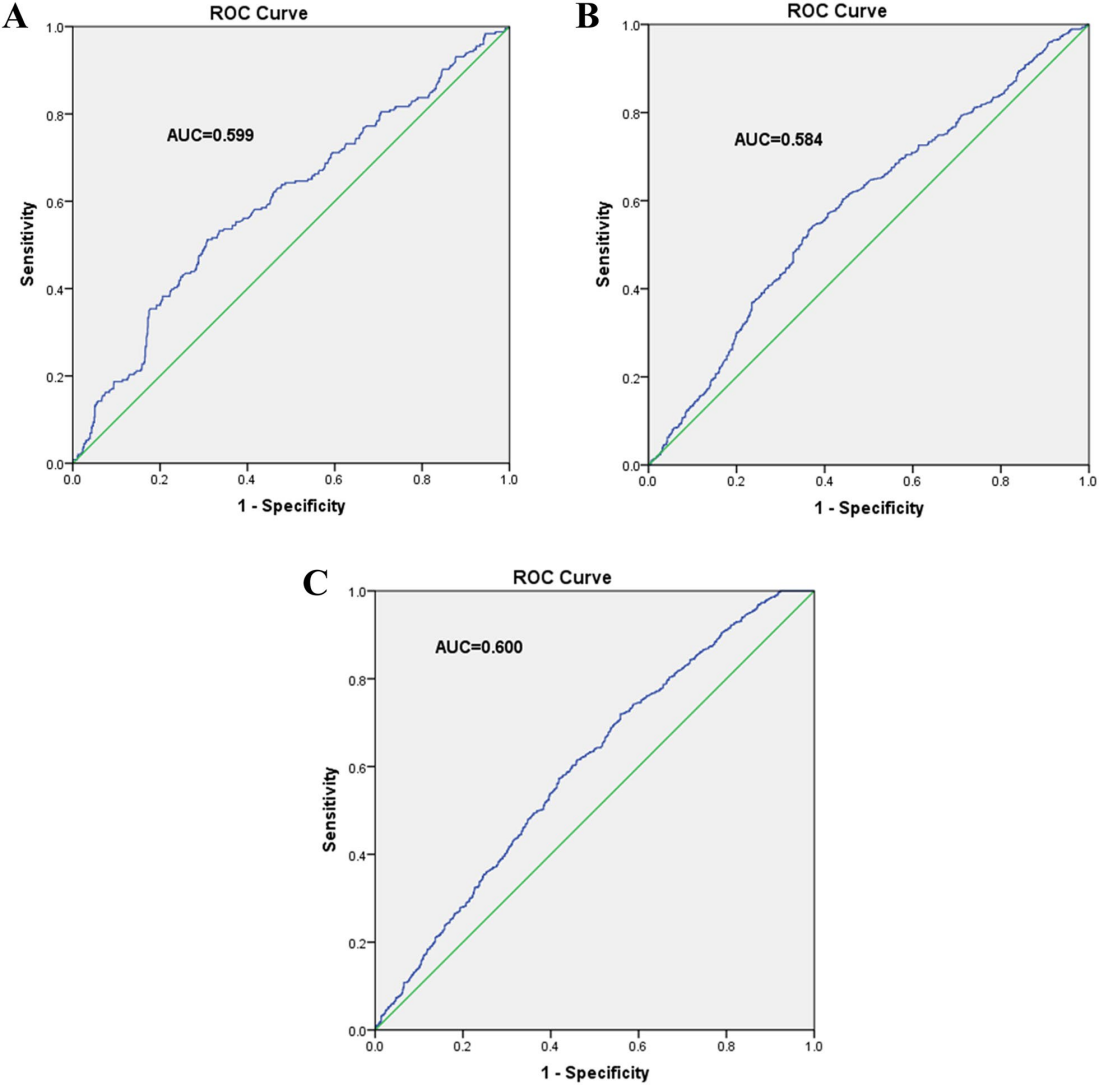
ROC Curve of cortisol and risk of albuminuria (**A**), DR (**B**) and DPN (**C**)

The clinical characteristics of diabetic patients grouped according to the level of microalbuminuria were summarized in Table 1. Microalbuminuria was found in 17.9% and macroalbuminuria was found in 13.9%. Compared with the microalbuminuria group, the duration of diabetes in patients with high microalbuminuria was significantly longer, and the levels of BMI, HbA1c, SBP, DBP, cortisol, sUA and blood lipids were higher and eGFR was lower (*p* < 0.05). The results of logistic regression analysis were listed in Table 2. Cortisol levels were stratified into Q1-Q4 according to the quartile. The first quartile (Q1) represented the lowest quartile and the fourth quartile (Q4) represented the highest quartile. When adjusted according to sex, age, BMI, WHR, duration of T2DM, HbA1c, BP, LDL-c, TG, TC, FFA, sUA and eGFR, the OR (95% CI) of microalbuminuria and macroalbuminuria in the last quartile were 3.396(2.030,5.682) and 8.407(3.726,18.971) compared with the first quartile. This suggested that the positive correlation between cortisol and albuminuria risk was independent (*p* < 0.001). In addition, we used ROC curve analysis to assess the diagnostic performance of cortisol in order to estimate the risk of proteinuria in diabetic patients (Fig. 1A). The AUC of T2DM proteinuria based on cortisol was 0.599 (optimal cutoff value, 286.35 nmol/L; sensitivity, 51.2%; specificity, 69.2%; *p* < 0.001).

**Table 1:** Clinical characteristics of the urinary microalbumin and macroalbuminuria groups in type 2 diabetic patient

Variables	Normoalbuminuria	Microalbuminuria	Macroalbuminuria	*p*-value
N	940	246	192	< 0.001*
Gender(male), %	446 (47.4%)	126 (51.2%)	111 (57.8%)	0.028*
Age, y	62.1 ± 11.3	62.6 ± 12.9	62.7 ± 12.5	0.735
BMI, kg/m^2^	25.8 ± 3.5	26.1 ± 4.0	26.6 ± 4.3	0.025*
WHR	0.94 ± 0.07	0.97 ± 0.09	0.97 ± 0.07	< 0.001*
DM duration, y	10.3 ± 7.5	12.0 ± 8.2	14.8 ± 8.1	< 0.001*
HbA1c, %	7.7 (6.7,9.1)	8.8 (7.2,10.5)	8.9 (7.3,10.4)	< 0.001*
SBP, mmHg	136 ± 16	144 ± 18	153 ± 23	< 0.001*
DBP, mmHg	77 ± 11	80 ± 12	82 ± 12	< 0.001*
Cortisol	254 (219,288)	287 (238,326)	299 (246,373)	< 0.001*
eGFR, mL/min/1.73 m2	122.1 ± 40.9	114.1 ± 49.9	90.2 ± 57.8	< 0.001*
Lipid Profile, mmol/L				
LDL-c	2.61 ± 0.87	2.64 ± 0.89	3.15 ± 1.38	< 0.001*
FFA	0.39 ± 0.19	0.43 ± 0.19	0.42 ± 0.21	0.022*
TC	4.38 ± 1.07	4.44 ± 1.11	5.13 ± 1.81	< 0.001*
TG	1.34 (0.94,1.96)	1.46 (1.07,2.30)	1.71 (1.17,2.84)	< 0.001*
sUA, umol/L	310 (259,368)	319 (267,390)	374 (309,454)	< 0.001*
Smoking History	220 (23.4%)	68 (27.6%)	56 (29.2%)	0.137
Drinking History	216 (23.0%)	63 (25.6%)	51 (26.6%)	0.454

Kruskal–Wallis H test or Chi-square test

Normally distributed variables are expressed as mean ± standard deviation, non-normal variables are expressed as median (IQR) and categorical variables are expressed as percentage (%)

*BMI* body mass index, *BP* blood pressure, *DM* diabetes mellitus, *WHR* waist hip ratio, *FFA* free fatty acid, *HbA1c* glycated hemoglobin, *eGFR* epidermal growth factor receptor, *LDL-C* Low density lipoprotein cholesterol, *TC* total cholesterol, *TG* triglyceride, *sUA* serum uric acid, *IQR* inter-quartile range

**Table 2:** Adjusted odds ratios of the quartiles of cortisol levels for the microalbuminuria and macroalbuminuria in Chinese type 2 diabetic participants

Quartiles	Microalbuminuria		Macroalbuminuria	
	OR (95% CI)	*p*-value	OR (95% CI)	*p*-value
Q1(≤ 225.4)	–	–	–	–
Q2(225.4–263.1)	0.704 (0.392, 1.266)	0.242	2.010 (0.851, 4.7495)	0.112
Q3(263.1–303.2)	1.213 (0.715, 2.057)	0.475	1.655 (0.686, 3.989)	0.262
Q4(> 303.2)	3.396 (2.030, 5.682)	< 0.001*	8.407 (3.726, 18.971)	< 0.001*

Logistic regression analysis. Adjusted for sex, age, BMI, WHR, duration of T2DM, HbA1c, BP, LDL-c, TG, TC, FFA, sUA and eGFR

### Association of cortisol level with NPDR and PDR

Table 3 summarized the clinical characteristics of patients classified according to the stages of diabetic retinopathy. NPDR accounted for 48.2% and PDR for 4.4%. Compared with the group without retinopathy, the duration of diabetes in retinopathy group was significantly prolonged, the level of age, HbA1c, SBP and cortisol were higher and TG, BMI, DBP, eGFR was lower (*p* < 0.05). Table 4 listed the results of logistic regression analysis. After adjusting for age, BMI, duration of T2DM, HbA1c, BP, TG and eGFR, the OR (95% CI) of NPDR and PDR in the last quartile was 2.007 (1.401, 2.875) and 7.122 (2.525, 20.090) compared with the first quartile. This suggested that the positive correlation between cortisol and DR risk is independent (*p* < 0.001). The analysis of the ROC curve of cortisol and risk of diabetic retinopathy is shown in Fig. 1B. AUC based on cortisol for predicting diabetic retinopathy was 0.584 (optimal cutoff value, 296.95 nmol/L; sensitivity, 54.4%; specificity, 62.6%; *p* < 0.001).

**Table 3:** Clinical characteristics of NPDR and PDR in participants

Variables	NDR	NPDR	PDR	*p*-value
N	644	653	59	< 0.001*
Gender(male), %	347 (53.9%)	312 (47.8%)	31 (52.5%)	0.086
Age, y	58.9 ± 12.3	62.7 ± 10.6	60.58 ± 12.1	< 0.001*
BMI, kg/m^2^	26.2 ± 4.0	25.3 ± 3.3	24.5 ± 3.9	< 0.001*
WHR	0.95 ± 0.07	0.96 ± 0.07	0.95 ± 0.07	0.051
DM duration, y	8.7 ± 6.6	12.4 ± 7.8	12.7 ± 8.2	< 0.001*
HbA1c, %	7.9 (6.8,9.5)	8.3 (7.0,9.7)	8.4 (7.2,9.6)	0.035*
SBP, mmHg	136 ± 17	140 ± 19	145 ± 24	< 0.001*
DBP, mmHg	79 ± 11	78 ± 11	78 ± 11	0.046*
Cortisol	269 (227,332)	305 (244,369)	327 (277,395)	< 0.001*
eGFR, mL/min/1.73 m2	132.3 ± 65.4	112.2 ± 51.5	118.9 ± 59.0	< 0.001*
Lipid Profile, mmol/L				
LDL-c	2.77 ± 0.86	2.69 ± 1.03	2.51 ± 1.23	0.083
FFA	0.39 ± 0.18	0.40 ± 0.21	0.37 ± 0.16	0.430
TC	4.52 ± 1.04	4.52 ± 1.31	4.22 ± 1.46	0.216
TG	1.41(1.00,2.12)	1.34(0.95,2.00)	1.17(0.88,1.60)	0.005*
sUA, umol/L	322 (264,390)	320 (263,385)	300 (253,360)	0.105
Smoking History	181 (28.1%)	162 (24.8%)	15 (25.4%)	0.652
Drinking History	172 (26.7%)	160 (24.5%)	14 (23.7%)	0.397

Kruskal–Wallis H test or Chi-square test

Normally distributed variables are expressed as mean ± standard deviation, non-normal variables are expressed as median (IQR) and categorical variables are expressed as percentage (%)

*BMI* body mass index, *BP* blood pressure, *DM* diabetes mellitus, *WHR* waist hip ratio, *FFA* free fatty acid, *HbA1c* glycated hemoglobin, *eGFR* epidermal growth factor receptor, *LDL-C* Low density lipoprotein cholesterol, *TC* total cholesterol, *TG* triglyceride, *sUA* serum uric acid, *IQR* inter-quartile range

**Table 4:** Adjusted odds ratios of the quartiles of cortisol levels for the NPDR and PDR in type 2 diabetic participants

Quartiles	NPDR		PDR	
	OR (95% CI)	*p*-value	OR (95% CI)	*p*-value
Q1(≤ 234.3)	–	–	–	–
Q2(234.3–284.3)	1.222 (0.866, 1.724)	0.253	4.340 (1.515, 12.436)	0.006
Q3(284.3–356.7)	1.775 (1.253, 2.516)	0.001	3.350 (1.114, 10.069)	0.031
Q4(> 356.7)	2.007 (1.401, 2.875)	< 0.001*	7.122 (2.525, 20.090)	< 0.001*

Logistic regression analysis. Adjusted for age, BMI, duration of T2DM, HbA1c, BP, TG and eGFR

### Association of cortisol level with NDPN and DPN

Table 5 described the clinical characteristics of patients with diabetic neuropathy. Among them, DPN accounts for 70.5%. Compared with group NDPN, the duration of diabetes in group DPN was significantly prolonged, the age was greater, the level of HbA1c and cortisol increased, and the level of eGFR was lower (*p* < 0.05). The result of logistic regression analysis was shown in Table 6. After adjusting for age, BMI, WHR, T2DM course, HbA1c, blood pressure, smoking and drinking rate, blood lipid and EGFR, the OR (95% CI) of DPN in the last quartile was 1.956 (1.371, 2.792) and that in the third quartile was 1.854 (1.319, 2.608). This suggests that the positive correlation between cortisol and DPN risk is independent (*p* < 0.001). The subject ROC analysis of cortisol and DPN risk is shown in Fig. 1C. The AUC of DPN based on cortisol was 0.600 (optimal cutoff value, 272.75 nmol/L; sensitivity, 71.9%; specificity, 44.1%; *p* < 0.001).

**Table 5:** Clinical Characteristics of NDPN and DPN in participants

Variables	NDPN	DPN	*p*-value
N	657	1572	–
Gender(male), %	348 (53.0%)	851 (54.1%)	0.614
Age, y	58.6 ± 12.7	64.4 ± 10.6	< 0.001*
BMI, kg/m^2^	26.3 ± 3.6	26.0 ± 3.5	0.030
WHR	0.95 ± 0.07	0.95 ± 0.07	0.297
DM duration, y	8.3 ± 6.7	12.4 ± 7.5	< 0.001*
HbA1c, %	8.0 (6.8,9.4)	8.1 (7.0,9.5)	0.152
SBP, mmHg	140 ± 18	141 ± 19	0.892
DBP, mmHg	81 ± 11	78 ± 12	< 0.001*
Cortisol	287 (226, 260)	322 (263, 393)	< 0.001*
eGFR, mL/min/1.73 m2	133.8 ± 58.9	121.2 ± 52.1	0.002
Lipid Profile, mmol/L			
FFA	0.44 ± 0.21	0.42 ± 0.21	0.026
TC	4.60 ± 1.25	4.55 ± 1.21	0.500
TG	1.47 (1.02, 2.17)	1.37 (0.96,2.03)	0.018
Smoking History	156 (23.7%)	456 (29.0%)	0.011
Drinking History	173 (26.3%)	442 (28.1%)	0.390

Kruskal–Wallis H test or Chi-square test

Normally distributed variables are expressed as mean ± standard deviation, non-normal variables are expressed as median (IQR) and categorical variables are expressed as percentage (%)

*BMI* body mass index, *BP* blood pressure, *DM* diabetes mellitus, *WHR* waist hip ratio, *FFA* free fatty acid, *HbA1c* glycated hemoglobin, *eGFR* epidermal growth factor receptor, *LDL-C* Low density lipoprotein cholesterol, *TC* total cholesterol, *TG* triglyceride, *sUA* serum uric acid, *IQR* inter-quartile range

**Table 6:** Adjusted odds ratios of the quartiles of cortisol levels for the DPN in type 2 diabetic participants

Quartiles	DPN	
	OR (95% CI)	*p*-value
Q1(≤ 251.2)	–	–
Q2(251.2–311.6)	1.461 (1.054, 2.026)	0.023
Q3(311.6–385.2)	1.854 (1.319, 2.608)	< 0.001*
Q4(> 385.2)	1.956 (1.371, 2.792)	< 0.001*

Logistic regression analysis. Adjusted for sex, age, BMI, WHR, duration of T2DM, HbA1c, BP, smoking and drinking rate, TG, TC, FFA and eGFR

## Discussion

In this retrospective study, we evaluated the relationship between cortisol levels and diabetic microangiopathy in adults with T2DM in Shandong province. The results showed that the prevalence of microvascular complications was higher in diabetic patients with higher cortisol levels. Elevated cortisol is positively correlated with risk of macroalbuminuria, DR and DPN, independent of age, duration of diabetes or other metabolic factors. ROC analysis revealed that the optimal cutoff point of cortisol for the prevalence of DKD, DR and DPN was 286.35 nmol/L, 296.95 nmol/L and 272.75 nmol/L in patients with T2DM. That means, patients with T2DM may be at increased risk of microvascular complications with cortisol levels in the range of 272.75–296.95 nmol/L or more. Therefore, cortisol may be a potential metabolic marker for diabetic microangiopathy, which needed further study. Up to present, our study has evaluated the prevalence of diabetic microvascular complications for the first time, and highlighted the relationship between cortisol levels and different diabetic microvascular complications.

### Cortisol and diabetes mellitus

T2DM is a major and growing epidemic lifestyle disease. Microvascular complications such as DR, DKDN, and DN are the major long-term complications increasing in parallel, affecting approximately 30% of patients with type 1 diabetes mellitus (T1DM) and 40% of patients with T2DM [16]. Glucocorticoid secretion was thought to be a possible link between insulin resistance and metabolic syndrome (hypertension, obesity, coronary heart disease, hyperlipidemia and type two diabetes) in patients with type 2 diabetes [17, 18]. Cortisol increased insulin resistance and induces type 2 diabetes through activation of lipolysis and free fatty acid release [19]. In fact, studies have shown that subclinical Cushing syndrome also increases the risk of type 2 diabetes [20]. In recent years, the relationship between type 2 diabetes and hypothalamus pituitary adrenal axis has been extensively studied. Many studies have shown that the levels of serum cortisol and late-night salivary cortisol in diabetic subjects are significantly higher after morning and low dose dexamethasone tests [21–23]. In contrast, some studies showed that cortisol was not associated with diabetes risk in Mendel’s genetic prediction [24], and salivary cortisol was gender related [25]. Therefore, the relationship between hypothalamus pituitary adrenal axis and diabetes risk was controversial.

In the past few years, few studies have confirmed the association between cortisol and diabetic microvascular complications. Our study will provide a new insight into the relationship between these two factors, that is, cortisol may be a potential clinical marker for early identification of diabetic microvascular complications. The relationship between cortisol and different complications will be discussed below.

### Cortisol and albuminuria

Similar to our findings, previous clinical studies confirmed cortisol and microalbuminuria in diabetic patients [9]. Koh et al. found that urinary albumin excretion increased in more than 80% of patients with Cushing's syndrome and reversed after treatment [26]. The earliest explanation was that the increase of urinary albumin in Cushing's syndrome might be the result of abnormal lipid metabolism. Glucocorticoids could lead to the increase of visceral fat content, which in turn leads to the increase of plasma free fatty acid (FFA) concentration. Excessive FFA would bind to serum albumin and increased the excretion of albumin into the urine. The increase of FFA might also activate the protein kinase-C (PKC) pathway in renal vascular endothelial cells, which has been proved to play an important role in the pathogenesis of diabetic nephropathy [27–29]. Our findings showed higher cortisol and lipid levels in patients with nephropathy, suggesting that cortisol may contribute to the development of nephropathy by stimulating increased FFA synthesis. Janssen and other scholars proposed that microalbuminuria is more common in patients with cortisol balance disorder, which may be related to endothelial injury. The cortisol level after low-dose dexamethasone test was positively correlated with high-sensitive C-reactive protein (hsCRP) and GGT. Microalbuminuria and elevated hsCRP levels were known predictors of early endothelial injury [30]. In addition, diabetic subjects with chronic complications might be exposed to chronic stress, which lead to increased cortisol secretion [31].

### Cortisol and DR

Several recent clinical studies have described the relationship between cortisol and the severity of diabetic retinopathy [10, 32]. Cortisol levels were positively correlated with central subfield thickness (CST), cube average thickness (CAT) and cube volume (CV), which represented the severity of DR. The possible mechanism of cortisol in DR was hyperglycemia and insulin resistance. Hyperglycemia could activate PKC pathway and increase the formation of advanced glycation end products, resulting in oxidative stress [33, 34]. In addition, the activation of PKC pathway lead to the activation of mitogen activated protein kinase (MAPK) and the phosphorylation of some major transcription factors, which also increase the gene expression of various stress-related components. The activation of MAPK, as a signal of vascular endothelial growth factor (VEGF), changed the permeability of retinal capillaries and leaded to the formation of DR [3]. We also found that the severity of DR was associated with increased cortisol levels, which might be attributed to increased glycosylation products as a result of PKC pathway activation.

### Cortisol and DPN

Previous clinical studies have shown that cortisol levels in patients with multiple diabetic neuropathy are elevated, compared with those without diabetic neuropathy. Moreover, cortisol levels were higher in patients with painful diabetic neuropathy [35]. Chiodini et al. studied 59 asymptomatic diabetic neuropathy patients. They found that the HPA axis activity of the patients was increased than that of the normal subjects [36]. The possible and reasonable mechanism was that catecholamine activates HPA pathway. It is well known that catecholamine can stimulate the secretion of corticotropin releasing hormone in hypothalamic neurons, while cholinergic tension can inhibit the secretion of corticotropin releasing hormone [37, 38]. The demyelination and axonal deletion of hypothalamic neurons in diabetic patients may increase the electrogenic properties of injured and regenerating neurons, which provide a reasonable mechanism for axis activation [35]. Animal experiments showed that excessive glucocorticoid can destroy the structure, function and vitality of brain cells [39]. Exposure to excessive glucocorticoids can lead to changes in neural plasticity, decreased neurogenesis, loss of hippocampal cells and changes in oligodendrocyte mediated remyelination [40–42]. In addition, cortisol induced the loss of myelin sheath around axons in postnatal rats [43]. As everyone knows, the pathogenesis of diabetic neuropathy was related to irreversible damage of neurons and glial cells. Therefore, excessive cortisol may change the microstructure of white matter and lead to nerve demyelination [44]. This is consistent with our findings that DPN is associated with elevated cortisol levels, which may be caused by HPA axis activation.

In this study, we found that cortisol was positively correlated with the severity of diabetic microangiopathy. Multiple logistic regression analysis further confirmed that corticosteroid remained an independent risk factor for diabetic microangiopathy after adjusting for other clinical factors (duration of diabetes, HbA1c, SBP, blood lipids, etc.). Cortisol may play a role in vascular endothelial injury, promoting albuminuria excretion, oxidative stress and activating MAPK pathway. The related pathophysiological mechanisms still need to be further explored.

This study has several limitations that need to be explained. First, this retrospective study cannot infer causality. Secondly, all recruited patients were hospitalized, so the results could not represent other parts of China. Third, there is not enough experimental evidence to explain the relationship between them.

## Conclusion

Above all, our study showed that high levels of cortisol were significantly associated with the risk of diabetic microangiopathy. Patients with T2DM may be at increased risk of microvascular complications with cortisol levels in the range of 272.75–296.95 nmol/L or more. Therefore, cortisol may be a potential metabolic marker for diabetic microangiopathy. The relationship between cortisol and diabetic microangiopathy will open up a new area of research, and prospective cohort studies are needed to identify causal relationships.

## Data Availability

The data used to support the findings of this study are available from the corresponding author upon request.

## References

[1] Thomas MC, Brownlee M, Susztak K, Sharma K, Jandeleit-Dahm KA, Zoungas S, Rossing P, Groop PH, Cooper ME (2015). Diabetic kidney disease. Nat Rev Dis Primers.

[2] Tuttle KR, Agarwal R, Alpers CE, Bakris GL, Brosius FC, Kolkhof P, Uribarri J (2022). Molecular mechanisms and therapeutic targets for diabetic kidney disease. Kidney Int.

[3] Cheung N, Mitchell P, Wong TY (2010). Diabetic retinopathy. Lancet.

[4] Kropp M, Golubnitschaja O, Mazurakova A, Koklesova L, Sargheini N, Vo TKS, de Clerck E, Polivka J, Potuznik P, Polivka J (2023). Diabetic retinopathy as the leading cause of blindness and early predictor of cascading complications-risks and mitigation. EPMA J.

[5] Lambertsen KL, Finsen B, Clausen BH (2019). Post-stroke inflammation-target or tool for therapy?. Acta Neuropathol.

[6] Reynolds RM, Labad J, Strachan MW, Braun A, Fowkes FG, Lee AJ, Frier BM, Seckl JR, Walker BR, Price JF (2010). Elevated fasting plasma cortisol is associated with ischemic heart disease and its risk factors in people with type 2 diabetes: the Edinburgh type 2 diabetes study. J Clin Endocrinol Metab.

[7] Steffensen C, Pereira AM, Dekkers OM, Jørgensen JO (2016). Diagnosis of endocrine disease: prevalence of hypercortisolism in type 2 diabetes patients: a systematic review and meta-analysis. Eur J Endocrinol.

[8] Steffensen C, Dekkers OM, Lyhne J, Pedersen BG, Rasmussen F, Rungby J, Poulsen PL, Jørgensen JOL (2019). Hypercortisolism in newly diagnosed Type 2 diabetes: a prospective study of 384 newly diagnosed patients. Hormone Metabol Res Hormon und Stoffwechselforschung Hormones et Metabolisme.

[9] Zhang X, Deng X, Zhou J, Qiu K, Deng M, Lin Z, Mosha SS, Li W (2020). The association of serum cortisol level with microalbuminuria in patients with type 2 diabetes and prediabetes. Int J Med Sci.

[10] Mohan A, Saxena S, Kaur A, Ali W, Akduman L (2021). Serum cortisol is a biomolecular biomarker for severity of diabetic retinopathy. Mol Vis.

[11] Wilkinson CP, Ferris FL, Klein RE, Lee PP, Agardh CD, Davis M, Dills D, Kampik A, Pararajasegaram R, Verdaguer JT (2003). Proposed international clinical diabetic retinopathy and diabetic macular edema disease severity scales. Ophthalmology.

[12] Inker LA, Astor BC, Fox CH, Isakova T, Lash JP, Peralta CA, Kurella Tamura M, Feldman HI (2014). KDOQI US commentary on the 2012 KDIGO clinical practice guideline for the evaluation and management of CKD. Am J Kidney Dis.

[13] Levey AS, de Jong PE, Coresh J, El Nahas M, Astor BC, Matsushita K, Gansevoort RT, Kasiske BL, Eckardt KU (2011). The definition, classification, and prognosis of chronic kidney disease: a KDIGO controversies conference report. Kidney Int.

[14] Pop-Busui R, Boulton AJ, Feldman EL, Bril V, Freeman R, Malik RA, Sosenko JM, Ziegler D (2017). Diabetic neuropathy: a position statement by the american diabetes association. Diabetes Care.

[15] Classification and Diagnosis of Diabetes (2021). Standards of medical care in diabetes-2021. Diabetes Care.

[16] Pelluri R, Srikanth K, Chimakurthy J, Nagasubramanian VR (2021). Microvascular complications and their associated risk factors among rural Type 2 diabetic population: a cross-sectional study. SN Comprehensive Clin Med.

[17] Chiodini I, Adda G, Scillitani A, Coletti F, Morelli V, Di Lembo S, Epaminonda P, Masserini B, Beck-Peccoz P, Orsi E (2007). Cortisol secretion in patients with type 2 diabetes: relationship with chronic complications. Diabetes Care.

[18] Vega-Beyhart A, Iruarrizaga M, Pané A, García-Eguren G, Giró O, Boswell L, Aranda G, Flores V, Casals G, Alonso C (2021). Endogenous cortisol excess confers a unique lipid signature and metabolic network. J Mol Med (Berl).

[19] Anagnostis P, Athyros VG, Tziomalos K, Karagiannis A, Mikhailidis DP (2009). Clinical review: the pathogenetic role of cortisol in the metabolic syndrome: a hypothesis. J Clin Endocrinol Metab.

[20] Chiodini I, Torlontano M, Scillitani A, Arosio M, Bacci S, Di Lembo S, Epaminonda P, Augello G, Enrini R, Ambrosi B (2005). Association of subclinical hypercortisolism with type 2 diabetes mellitus: a case-control study in hospitalized patients. Eur J Endocrinol.

[21] Ortiz R, Kluwe B, Odei JB, Echouffo Tcheugui JB, Sims M, Kalyani RR, Bertoni AG, Golden SH, Joseph JJ (2019). The association of morning serum cortisol with glucose metabolism and diabetes: the Jackson Heart Study. Psychoneuroendocrinology.

[22] Hepsen S, Sencar E, Sakiz D, Akhanli P, Ucan B, Unsal I, Ozbek M, Cakal E (2020). Serum cortisol level after low dose dexamethasone suppression test may be predictive for diabetes mellitus and hypertension presence in obese patients: a retrospective study. Diabetes Res Clin Pract.

[23] Johar H, Emeny RT, Bidlingmaier M, Kruse J, Ladwig KH (2016). Sex-related differences in the association of salivary cortisol levels and type 2 diabetes. Findings from the cross-sectional population based KORA-age study. Psychoneuroendocrinology.

[24] Kwok MK, Kawachi I, Rehkopf D, Schooling CM (2020). The role of cortisol in ischemic heart disease, ischemic stroke, type 2 diabetes, and cardiovascular disease risk factors: a bi-directional Mendelian randomization study. BMC Med.

[25] Champaneri S, Xu X, Carnethon MR, Bertoni AG, Seeman T, Diez Roux A, Golden SH (2012). Diurnal salivary cortisol and urinary catecholamines are associated with diabetes mellitus: the Multi-Ethnic Study of Atherosclerosis. Metab Clin Exp.

[26] Koh JM, Kim JY, Chung YE, Park JY, Shong YK, Hong SK, Kim GS, Lee KU (2000). Increased urinary albumin excretion in Cushing's syndrome: remission after correction of hypercortisolaemia. Clin Endocrinol.

[27] Björntorp P (1991). Metabolic implications of body fat distribution. Diabetes Care.

[28] Babazono T, Kapor-Drezgic J, Dlugosz JA, Whiteside C (1998). Altered expression and subcellular localization of diacylglycerol-sensitive protein kinase C isoforms in diabetic rat glomerular cells. Diabetes.

[29] Dubois D, Chanson P, Timsit J, Chauveau D, Nochy D, Guillausseau PJ, Lubetzki J (1994). Remission of proteinuria following correction of hyperlipidemia in NIDDM patients with nondiabetic glomerulopathy. Diabetes Care.

[30] Janssen SF, Twickler TB, Jublanc C, Cramer MJ, Bruckert E (2008). Patients with the metabolic syndrome and a disturbed cortisol balance display more microalbuminuria. Diab Vasc Dis Res.

[31] Richardson AP, Tayek JA (2002). Type 2 diabetic patients may have a mild form of an injury response: a clinical research center study. Am J Physiol Endocrinol Metab.

[32] Mohan A, Saxena S, De S, Kaur A, Ali W, Gilhotra JS, Meyer CH (2021). Increased serum cortisol is associated with alterations in cross-sectional and topographic OCT parameters in diabetic retinopathy: a preliminary study. Int Ophthalmol.

[33] Brownlee M (2001). Biochemistry and molecular cell biology of diabetic complications. Nature.

[34] Geraldes P, King GL (2010). Activation of protein kinase C isoforms and its impact on diabetic complications. Circ Res.

[35] Tsigos C, Young RJ, White A (1993). Diabetic neuropathy is associated with increased activity of the hypothalamic-pituitary-adrenal axis. J Clin Endocrinol Metab.

[36] Chiodini I, Di Lembo S, Morelli V, Epaminonda P, Coletti F, Masserini B, Scillitani A, Arosio M, Adda G (2006). Hypothalamic-pituitary-adrenal activity in type 2 diabetes mellitus: role of autonomic imbalance. Metab Clin Exp.

[37] Füzesi T, Wittmann G, Liposits Z, Lechan RM, Fekete C (2007). Contribution of noradrenergic and adrenergic cell groups of the brainstem and agouti-related protein-synthesizing neurons of the arcuate nucleus to neuropeptide-y innervation of corticotropin-releasing hormone neurons in hypothalamic paraventricular nucleus of the rat. Endocrinology.

[38] Llorente I, Lizcano F, Alvarez R, Diez N, Sopena M, Gil MJ, Salvador J (1996). Cholinergic modulation of spontaneous hypothalamic-pituitary-adrenal activity and its circadian variation in man. J Clin Endocrinol Metab.

[39] Sapolsky RM, Krey LC, McEwen BS (1986). The neuroendocrinology of stress and aging: the glucocorticoid cascade hypothesis. Endocr Rev.

[40] Goosens KA, Sapolsky RM, Riddle DR (2007). Frontiers in neuroscience stress and glucocorticoid contributions to normal and pathological aging. Brain aging: models, methods, and mechanisms.

[41] Alonso G (2000). Prolonged corticosterone treatment of adult rats inhibits the proliferation of oligodendrocyte progenitors present throughout white and gray matter regions of the brain. Glia.

[42] Miyata S, Koyama Y, Takemoto K, Yoshikawa K, Ishikawa T, Taniguchi M, Inoue K, Aoki M, Hori O, Katayama T (2011). Plasma corticosterone activates SGK1 and induces morphological changes in oligodendrocytes in corpus callosum. PLoS ONE.

[43] Bohn MC, Friedrich VL (1982). Recovery of myelination in rat optic nerve after developmental retardation by cortisol. J Neurosci Off J Soc Neurosci.

[44] Pires P, Santos A, Vives-Gilabert Y, Webb SM, Sainz-Ruiz A, Resmini E, Crespo I, de Juan-Delago M, Gómez-Anson B (2015). White matter alterations in the brains of patients with active, remitted, and cured cushing syndrome: a DTI study. AJNR Am J Neuroradiol.

